# The Imperial Cancer Research Fund

**Published:** 1907-07-13

**Authors:** 


### THE IMPERIAL CANCER RESEARCH FUND.

The annual meeting of the General Committee of the Imperial Cancer Research Fund was held at Marlborough House on Monday, July 1st, and the report of the proceedings shows that steady progress is being made in the systematic investigation of the many problems which must be solved before the cause of cancer can be discovered. Not the least formidable among these problems is the necessity for controlling the results obtained by various reputed cures for the disease. It is only right that these cases should be submitted to the test of experiment at the hands of independent workers. There is always a possibility that one of them may be found to possess the properties of cure or alleviation which in each case is claimed for it by the authors, and that a short cut to success may thus be obtained. So far the hope has been disappointed, but when the failure is announced by those who are occupying a purely scientific position it is impossible for the cancer curer to denounce the verdict as the result of an odium medicum which, they say, is similar to the odium theologicum of former days.

### The Trypsin Treatment.

The report states that:—

Serious attention had been given to the additional alleged cancer cures which had been brought to their notice during the past year. Unfortunately, it was impossible to assign a curative value to any of them. It was desirable from a public point of view to allude specifically to one alleged remedy for cancer which had been tested last year and had again been subjected to renewed tests. This alleged remedy —trypsin alone or in conjunction with amylopsin or as pancreatic extract, in which trypsin, as such, was absent—had received a quasi-scientific basis by the assertion that its employment had cured two mice inoculated with Jensen's carcinoma. When the assertion was made, its validity was carefully tested on mice with rapidly-growing tumours. The large number of observations then made showed that the cures claimed were based on fallacies inseparable from so small a number of experiments. The reinvestigation of this alleged remedy, with the modifications since suggested, had shown that it was incapable of curing mice of inoculated cancer or of influencing the progressive growth of tumours.

The alleged remedy has failed as completely in practice as it has done in the laboratory, and this official declaration should stop effectually the further use of trypsin in the treatment of cancer by those who are endeavouring honestly to give their patients the best advice.

### Cancer Mortality.

The report deals this year with two interesting factors in the cancer mortality, the liability of the individual and the liability of the family; in other words, the death-rate in males and females, and the question of heredity. In 1889 the Registrar-General stated that 1 out of 21 men and 1 out of 12 women reaching the age of 35 eventually died of cancer. In 1905 the Registrar-General, using the same methods, calculates that 1 in 12 men and 1 in 8 women attaining the age of 35 will eventually die of cancer. The chances of dying of cancer, therefore, are approximating in the two sexes and are becoming more numerous for everyone, though proportionately more men succumb to this disease now than formerly. Indeed, the frequency of cancer as a cause of death is so great that few families of any size escape.

### Cancer Incidence.

The average human life is too long to allow of detailed analyses of the incidence of cancer in a large number of families, but the difficulty is overcome by experimenting with such short-lived and fruitful animals as the mouse. The report continues:—

In the mouse it was now in a fair way to a definite settlement by means of breeding and in-breeding experiments on a large scale. As in a man, so in the mouse, the total number of cases of cancer occurring in different strains varied. The disease had been so frequent as to lead some observers to assert the occurrence of epidemics in certain cages. Their prolonged investigations had given no support to this interpretation of the apparent greater frequency of cancer in some communities of mice as compared with others. The surgical removal of spontaneously-occurring tumours had enabled them to prolong the life of many mice and to breed from them. In that way they had obtained mice of a known cancerous parentage. By successively crossing other spontaneously affected animals with the offspring of cancerous parents, strains were being obtained in which the cancerous heredity was $\frac{1}{2}$, $\frac{3}{4}$, or $\frac{15}{16}$, and even higher. This concentration of a hypothetical hereditary factor in a known amount, and in large numbers of animals of known age, should in the course of a few years definitely settle whether there was a family or only an individual liability to the disease. At the same time mice of known cancerous stock might be indispensable to further attempts to produce cancer experimentally—for example, by the artificial application of external agencies having a mediate relation to its spontaneous appearance. All attempts to produce cancer experimentally had thus far failed in animals chosen at random. They had frequently pointed out that the origin of cancer was something entirely different from its continued growth either in the spontaneously attacked or in inoculated animals.

### Inoculated Cancer.

The resemblance between the continual growth of spontaneous and of inoculated cancer is, however, very close, but the origin of cancer must be studied in spontaneous cases. The experiments yield evidence that the growth of cancer consists of a succession of phases of increased and diminished energy of assimilation and growth. Perhaps the most significant part of the report relates the fact that mice which had been completely protected against the inoculation of cancer have developed the disease spontaneously. The methods for protection only prevented the grafts from taking, and did not hinder growth when once the grafts had taken and organic union with the part had been established. The fact that cancer develops spontaneously in animals completely protected against inoculation by grafts points to the probability that the organic union between the tumour and the host is established from the very beginning of the disease. Surgeons therefore do well to recommend the earliest possible removal of cancer to their patients, and it is possible from Dr. Bashford's work that an explanation will in due time be forthcoming as to the reason for the reappearance of cancer after many years of apparent immunity.

